# Minimally invasive application of botulinum toxin A in patients with idiopathic rhinitis

**DOI:** 10.1186/1746-160X-5-18

**Published:** 2009-10-16

**Authors:** Saskia Rohrbach, Katharina Junghans, Sibylle Köhler, Rainer Laskawi

**Affiliations:** 1 Department of Otolaryngology, Head and Neck Surgery, University of Göttingen, Germany; 2 Department of Audiology and Phoniatrics, Charite, Medical University of Berlin, Germany

## Abstract

**Background:**

Nasal hypersecretion due to idiopathic rhinitis can often not be treated sufficiently by conventional medication. Botulinum toxin A (BTA) has been injected into the nasal mucosa in patients with nasal hypersecretion with a reduction of rhinorrhea lasting for about 4 to 8 weeks. Since the nasal mucosa is well supplied with glands and vessels, the aim of this study was to find out if the distribution of BTA in the nasal mucosa and a reduction of nasal hypersecretion can also be reached by a minimally invasive application by sponges without an injection.

**Methods:**

Patients were randomly divided into two groups. The effect of BTA (group A, C, D) or saline as placebo (group B) was investigated in 20 patients with idiopathic rhinitis by applying it with a sponge soaked with BTA (40 units each nostril) or saline. Subgroups C and D contained these patients of group A and B who did not improve in symptoms one week after the original treatment (either BTA or saline) who then received the alternative medication. Changes of symptoms (rhinorrhea, nasal obstruction) were scored by the patients in a four point scale and counted (consumption of tissues, sneezing) in a diary. The patients were followed up weeks 1, 2, 4, 8 and 12.

**Results:**

There was a clear reduction of the amount of secretion in group A compared to group B, C and D. This did not correlate with the tissue consumption, which was comparably reduced in group A and B, but reduced less in group C and D. Sneezing was clearly reduced in group A but comparably unchanged in group B and C and increased in group D. Nasal congestion remained unchanged.

**Conclusion:**

In some patients with therapy-resistant idiopathic rhinitis BTA applied with a sponge is a long-lasting and minimal invasive therapy to reduce nasal hypersecretion.

## Background

Chronic rhinitis is a common condition affecting over 20% of the population [1]. Since patients with rhinitis form a heterogeneous group, until now there has been no universally accepted definition for the different entities. An attempt to take into consideration the pathophysiological mechanisms classified rhinitis in *allergic*, *infectious *and *other forms *[2]. *Other forms *include the idiopathic rhinitis (IR, also referred to as intrinsic, in former times vasomotor rhinitis), a diagnosis of exclusion which has not been as extensively investigated as allergic rhinitis [3]. Nevertheless nonallergic rhinitis may be just as common and disabling for the patient. Studies of prevalence of nonallergic rhinitis have reported that this ranges from around 20-50% amongst the rhinitis population [4,5]. All forms of rhinitis are caused by a permanent, convulsive or occasional nasal hyperreactivity. Nasal secretion and nasal patency is mainly controlled by the autonomic nervous system. Different neuropeptides participate in the complex innervation of the nasal system, among them vasoactive intestinal peptide (VIP), calcitonin gene-related peptide (CGRP), substance P (SP), nitric oxide (NO) and acetylcholine [6-10]. The discharge of excessive watery nasal fluids in allergic and non-allergic rhinitis is caused by an overactivity of the submucosal seromucinous glands and a massive exudation from the mucosal vasculature. All patients complain about the characteristic symptoms like nasal obstruction and sneezing, but especially rhinorrhea is the most obvious symptom to others and often the most bothering to the patient.

Therapeutic options in treating nasal hyperreactivity depend on the pathogenesis of the particular type of rhinitis and the current complaints of the patient. It includes allergen reduction, the application of local decongestants and topical steroids, specific immunotherapy or rhinosurgical treatment. Since few of the conventional treatments yield satisfactory results for reducing rhinorrhea irrespective how it is caused, further therapies have to be developed.

Botulinum toxin (BTX) is a neurotoxin that inhibits the release of acetylcholine from the presynaptic nerve terminal at the neuromuscular and neuroglandular junction [11]. It is therapeutically used in otorhinolaryngology for different dysfunctions like spasmodic dysphonia, dysphagia, oromandibular dystonia, and facial and cervical movement disorders. It has also been used in the autonomic nervous system to treat gustatory sweating [12,13] and sialorrhea [14,15]. Botulinum toxin type A (BTA) has been injected in the nasal mucosa in patients with IR and allergic rhinitis to reduce nasal fluids [16,17], having a stronger effect on the reduction of rhinorrhea than steroid injection [18].

Shaari et al. [19] demonstrated a decrease in experimentally induced rhinorrhea in dogs after placing gauze with BTA into the nasal cavity. BTA has been injected in the nasal mucosa of the lower and middle turbinate mucosa for allergic and IR by different authors, resulting in a significant and up to 8 weeks lasting reduction of nasal hypersecretion [16,17].

Earlier studies of our group, confirmed by others, showed a temporary degeneration of submucosal glands of the turbinate mucosa of guinea pigs after applying BTA with a sponge [20,21] and a significant reduction of rhinorrhea, tissue consumption and nasal obstruction in a female patient with IR after BTA treatment using the same method [22].

The aim of this study was to verify the results of the single case study in patients with IR by evaluating subjective symptomatic relief of rhinorrhea, nasal obstruction and sneezing and to observe the development of the number of tissues used after applying BTA minimally invasively with a sponge.

## Materials and methods

The study was approved by the ethic committee of the Georg-August University of Göttingen, in compliance with the Helsinki Declaration. All patients gave their written informed consent to participate.

**Figure 1:**
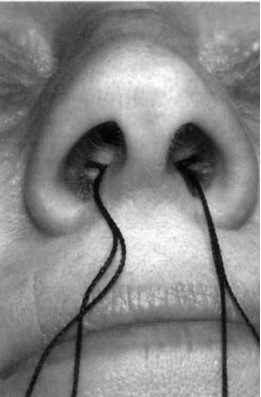
**Sponges placed in each nasal cavity**. The sponges are attached to a small thread for removal. Once they have contact to liquids, they expand and cover a large area of the mucosa of the nasal septum and the lower and middle turbinates.

Twenty patients with IR (5 female, 15 male, mean age 61.8 ± 10.0) were included into the study. Three patients did not finish the follow up time because of the long distance to our hospital and were excluded. Exclusion criteria were pregnancy or breast feeding, myasthenia, nasal anatomical abnormalities (septal deviation, polyps), acute infectious rhinosinusitis and simultaneous use of aminoglycosides. All patients had already a long history of IR with former treatment with decongestants, topical steroids or ipratropium bromide without effect. IR was diagnosed by means of history, clinical examination and negative skin prick test and in some patients by x-ray of the sinuses to exclude sinusitis. Patients were randomly divided into 2 groups:

In group A, 40 units of BTA (Botox^®^, Allergan Inc, Irvine, California; 1,6 ml = 40 units of BTX-A) were applied (total 80 units). In group B, the corresponding amount of 0.9% saline was used. Group C and D were these patients who did subjectively not note any reduction of symptoms (including all symptoms) one week after the treatment (either BTA or saline). Those patients were treated for a second time (other than first formula, group C = first treatment with BTA, second treatment with saline, group D = first treatment with saline, second treatment with BTA). The respective liquid (BTA or saline) was dropped on a sponge which expanded (Merocel^®^, Medtronic Xomed, Mystic, Connecticut, USA) after it was introduced in each nostril using bayonet forceps (see figure 1). This design of double treatment in some patients has been chosen to increase the compliance of patients. All patients included had the benefit to get BTA. The sponges stayed in the nose for 30 minutes and were then removed.

According to Kim et al. [16] we handed out a nose-diary in which the patients recorded the number of tissues used per day (each tissue was only used once), the number of sneezing per day and scored symptoms like nasal secretion and nasal congestion on a four-point scale (0 = no, 1 = mild, 2 = moderate, 3 = moderate to severe and 4 = severe), starting two weeks before the first treatment. The occurrence of a dry nose, smelling disorders or epistaxis was recorded daily. All patients were advised not to take any additional nasal therapy. The patients were followed up week 1, 2, 4, 8 and 12. The inspection of the nose diary, the patients' over all impression of the treatment and the clinical examination (anterior rhinoscopy) were done in every follow up and formed the basis for evaluating the study.

The sum of the severity of symptoms for each group was expressed as per cent of the original symptom severity before treatment (week -1 and -2 until time of first treatment).

## Results

The study groups were comprised as follows: group A (only BTA), n = 3 (1 female, 2 males; mean age 67,3 years; range 55-80 years); group B (only saline), n = 3 (3 males, mean age 71,3 years, range 59-80 years); group C (BTA/saline), n = 7 (2 females, 5 males, mean age 61,6 years, range 44-73 years); and group D (saline/BTA), n = 4 (2 females, 2 males, mean age 63,5 years, range 52-73 years). Three patients were lost for follow up because of the long distance to our hospital. Twelve of 17 (70.6%) patients realized the treatment, irrespective of what they received, as positive. The results for each patient can be seen in table 1.

**Table 1:** Overview on all groups after treatment

**group**	**n**	**patients**	**tissues**	**secretion**	**congestion**	**sneezing**
A	3	IN 3, 11, 16	▼+▼	▼▼▼	▼∅∅	▼▼▼

B	3	IN 6, 14, 15	▼▼▼	+▼▼	▼▼∅	▼▼▼

C	7	IN 1, 2, 4, 5, 10, 12, 17,	▼▼++ ▼▼▼	▼▼++ ▼▼▼	∅▼▼+ ∅∅▼	▼▼+▼ +▼▼

D	4	IN 7, 9, 13, 18	▼▼▼▼	+▼∅▼	∅∅∅▼	▼▼▼+

Results for all patients of group A-D concerning tissue consumption, nasal secretion, congestion and sneezing.**▼ **decreased,**∅ **no change,**+ **increased, IN = intrinsic rhinitis

The *tissue consumption *after 12 weeks in group A (only BTA) was reduced 42,57%, comparable in group B (only saline) 35,47%, in group C (BTA/saline) 27,24% and in group D (saline/BTA) 27,04%. The subjective scored *amount of secretion *was reduced in group A (only BTA) 46,39%, but in group B (only saline) 6,12%, in group C (BTA/saline) 24,70% and in group D (saline/BTA) 13,37%. The tissue consumption and the subjective scored amount of secretion did not correlate. Note the tissues used in group B compared to the amount of reduction of nasal secretion (reduction 35,47% versus 6,12% of reduction of nasal secretion). *Nasal congestion *was scored also and was reduced in group A (only BTA) 9,66%, in group B (only saline) 20,90%, in group C (BTA/saline) 27,72% and in group D (saline/BTA) 52,38% (n = 1).

**Figure 2:**
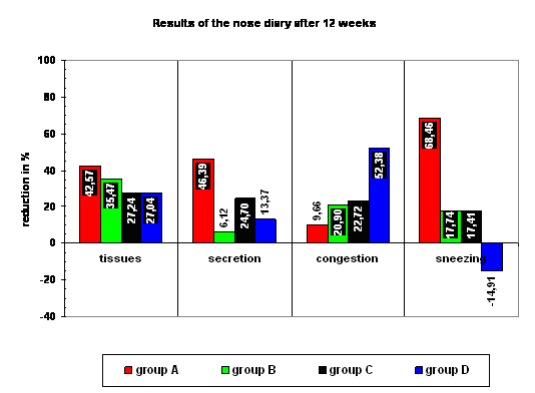
**Percentual reduction of tissue consumption, nasal secretion, nasal congestion and sneezing for group A-D 12 weeks after the treatment**. A clear decrease of nasal secretion and sneezing in group A (only BTA) is obvious. Note that group D shows an increase of sneezing 12 weeks after treatment.

**Figure 3:**
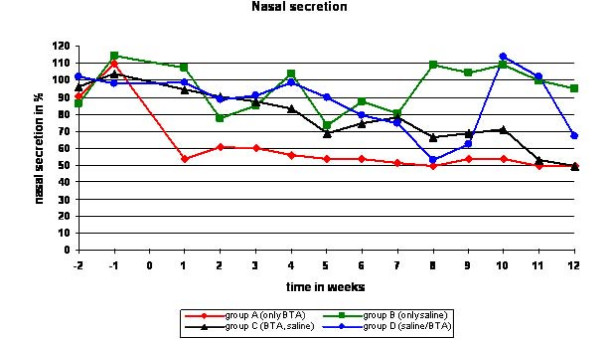
**Results of nasal secretion week -2 to week 12 for all groups in %**. Note the clear decrease of the amount of nasal secretion in group A (only BTA) compared to group B, C and D.

The symptom *"sneezing" *was reduced 68,46% in group A (only BTA), but only 17,74% in group B (only saline), 17,41% in group C (BTA/saline) and increased 14,91% in group D (saline/BTA) (see figure 2). The time courses for the reduction of nasal secretion can be seen in figure 3. A dry nose, smelling disorders or epistaxis did not occur in our patients.

## Discussion

The effect of BTA on glands has been described for different organs [16,19-23]. The way of its action in the nose has been postulated to be the inhibition of the release of acetylcholine from the pre-ganglion cholinergic nerve endings in the nasal mucosa, the inhibition of the release of acetylcholine from the pre-ganglion cholinergic nerve endings in the sphenopalatine ganglion and the induction of apoptosis of nasal glands [16,19,20].

However, not all glands seem to be influenced (group A, C and D). Shaari et al. [16] obtained an average decrease in rhinorrhea of 41% in dogs' experimentally induced rhinorrhea. One out of four dogs even showed an increase in secretion. Own results in adult guinea pigs nasal glands showed about 60% of degeneration after treatment with 40 units BTA (Botox^®^) with a sponge [20]. Apart from the fact that the area the toxin is able to reach after local application is restricted, the partial effect may be due to the abundance of other neuropeptides that are included in regulating the homeostasis of nasal secretion [24], but not influenced by an anticholinerg drug like BTA. On the other hand it is postulated that BTA also influences the effect of other neuropeptides in nasal secretion [6]. This might be the cause for the reduction of nasal secretion in some of our patients who did not show an effect after treatment with ipratropium bromide (anticholinerg) but who did show a reasonable effect after treatment with BTA. In some patients who were treated with BTA (patients of group C and D) we could not confirm a reasonable change in symptoms even though they were treated with BTA after they received saline first or after the treatment with BTA. We can not explain this phenomenon but we do not know the exact pathophysiological mechanism of IR, so in some patients acetylcholine might play a minor role in causing nasal hypersecretion. It seems as if we have to deal with a *BTA sensitive rhinitis*, which would be interesting to differentiate from other forms of nonallergic rhinitis.

We decided to introduce the toxin into the nose via small self-expanding sponges which, once having contact with the fluid, filled out the whole nasal cavity without letting any substance drop into the nasopharynx. Since the nasal mucosa is a very permeable tissue to absorb and excrete substances and since this method showed a serious effect on guinea pigs nasal glands, we carried out this minimally invasive and less painful method to reach a bigger area of nasal glands. Throughout the application time of 30 minutes, the patients could breathe through the mouth without any problems. The fact that not all patients who were treated reported an improvement is not restricted to our patients in this study, but is also known in patients which underwent BTA-injections into the nasal conchae to treat IR [16,17]. In contrast to the injection, by our method we do not exactly know which amount of BTA really reached the mucosa.

Before treating the patients, we had to decide which dose of BTA should be applied. Shaari et al. [19] used 50 units in soaked gauze to one nasal cavity in dogs. Others injected between 4 to 60 units BTA into the mucosa of the lower turbinates [16,25,26]. In guinea pigs and in one patient with IR, we applied 40 units BTA on a sponge [20,22]. To reach an intense and long-lasting effect even in patients with severe symptoms, we used 40 units per nasal cavity (total 80 units).

The symptom scores concerning the amount of secretion was clearly reduced in group A compared to group B, C and D. Interestingly, this did not correlate with the tissue consumption, which was comparably clearly reduced in group A and B, but reduced less in group C and D. One can speculate that the "use of tissues" intensely depends on the subjective assessment of patient and is therefore more than the other symptoms exposed to the patients' expectance. This study and other investigation on the influence of BTA on nasal secretion show that we need an objective indicator for the reduction of nasal secretion (i.e. with weighing a sponge introduced in the nose before a respective treatment and on a representative time after the treatment).

Sneezing was clearly reduced in group A but later and to a lesser degree in group B, C and D. Unal [17] described a significant reduction of sneezing in patients with allergic rhinitis after treatment with 40 or 60 units Botox^®^, whereas Kim et al. [16] did not report on a reduction in sneezing in patients with intrinsic rhinitis. Our findings might indicate an important role of acetylcholine as a relevant neurotransmitter in the sneezing reflex.

Most of our patients did not suffer from nasal congestion neither before nor after the treatment, which is more frequent in allergic rhinitis. The subjects have traditionally been classified as either "runners" (predominantly rhinorrhea) or "blockers" (predominantly nasal congestion), but many patients suffer from more than one symptom. Unal et al. [25], who treated patients with allergic rhinitis, showed a significant reduction in nasal congestion after injection of BTA. Nasal congestion in most of our patients of all groups did not change (only 1 patient in group D reported about reduction of nasal stuffiness), which confirms the results of Kim et al. [16] in patients with IR. Since nasal congestion is mainly regulated through the nasal vessels and not directly under the influence of acetylcholine, we did not expect a difference in nasal stuffiness. It is only imaginable that an improvement in nasal air flow occurs secondarily through a significant reduction of glandular volume.

Other groups who used BTA for nasal symptoms described a duration of effect for 4 to at least 8 weeks [16,25]. In our patients the reduction of all symptoms described, once occurred, lasted for at least 12 weeks. In adult guinea pigs, we could show a degeneration of submucosal glands after treatment with BTA applied with the same method (sponge). Regeneration was seen after 12 weeks [20]. The longer time of effect in those patients, who felt a reasonable reduction of rhinorrhea might be due to the dose but also to the application method we used, reaching an extended mucosal area with the possibility to block a maximum of nasal glands.

Even none of the patients reported about an increase in symptoms after treatment, some of the BTA treated subjects did not describe any improvement compared to other studies [16,25,26]. Since we do not know the exact pathophysiological mechanism of IR, in some patients acetylcholine might play a minor role in the origin of hypersecretion.

## Conclusion

This study could show that in some patients with IR, the minimally invasive application method of BTA with a sponge is a save, painless method which can lead to a long lasting reduction of nasal hypersecretion. Further studies should investigate methods to objectify the patients' symptoms, especially the amount of nasal secretion, and yield results in the question of dosages of BTA-application in the nose. A greater amount of patients could yield reliable results through statistical analysis.

## Competing interests

The authors declare that they have no competing interests.

## Authors' contributions

SR treated the patients, interpreted the results and drafted the manuscript. KJ and SK treated the patients and participated in constructing the tables.

RL conceived of the study, and participated in its design and coordination and helped to draft the manuscript. All authors read and approved the final manuscript.
